# Leaf hydraulic conductance declines in coordination with photosynthesis, transpiration and leaf water status as soybean leaves age regardless of soil moisture

**DOI:** 10.1093/jxb/eru380

**Published:** 2014-10-03

**Authors:** Anna M. Locke, Donald R. Ort

**Affiliations:** ^1^Department of Plant Biology, University of Illinois, Urbana, IL 61801, USA; ^2^Institute for Genomic Biology, University of Illinois, Urbana, IL 61801, USA; ^3^Global Change and Photosynthesis Research Unit, Agricultural Research Service, United States Department of Agriculture, Urbana, IL 61801, USA

**Keywords:** Development, drought, leaf age, leaf hydraulic conductance, leaf water potential, photosynthesis, senescence, stomatal conductance.

## Abstract

Photosynthesis requires sufficient water transport through leaves for stomata to remain open as water transpires from the leaf, allowing CO_2_ to diffuse into the leaf. The leaf water needs of soybean change over time because of large microenvironment changes over their lifespan, as leaves mature in full sun at the top of the canopy and then become progressively shaded by younger leaves developing above. Leaf hydraulic conductance (*K*_leaf_), a measure of the leaf’s water transport capacity, can often be linked to changes in microenvironment and transpiration demand. In this study, we tested the hypothesis that *K*_leaf_ would decline in coordination with transpiration demand as soybean leaves matured and aged. Photosynthesis (*A*), stomatal conductance (*g*_s_) and leaf water potential (Ψ_leaf_) were also measured at various leaf ages with both field- and chamber-grown soybeans to assess transpiration demand. *K*_leaf_ was found to decrease as soybean leaves aged from maturity to shading to senescence, and this decrease was strongly correlated with midday *A*. Decreases in *K*_leaf_ were further correlated with decreases in *g*_s_, although the relationship was not as strong as that with *A*. Separate experiments investigating the response of *K*_leaf_ to drought demonstrated no acclimation of *K*_leaf_ to drought conditions to protect against cavitation or loss of *g*_s_ during drought and confirmed the effect of leaf age in *K*_leaf_ observed in the field. These results suggest that the decline of leaf hydraulic conductance as leaves age keeps hydraulic supply in balance with demand without *K*_leaf_ becoming limiting to transpiration water flux.

## Summary

Short statement: Field and chamber studies show a decline in leaf hydraulic conductance as soybean leaves age that is independent of decreases in soil moisture.

## Introduction

Greater than 99% of the water absorbed by a plant’s roots is lost to the atmosphere through transpiration. This is an unavoidable consequence of allowing CO_2_ diffusion into leaves for photosynthesis, but it is also necessary for leaf cooling and plant nutrient uptake. Water requirements change over the plant’s and leaf’s lifespan, as fluctuating microenvironments around leaves alter transpiration demand on daily and seasonal timescales (Hinckley and Ritchie, 1970; Barrett *et al.*, 1996). Evapotranspiration and stomatal conductance are known to decrease as leaves age in many species (Constable and Rawson, 1980; Sobrado, 1994; Kositsup *et al.*, 2010). A plant’s carbon needs and photosynthetic capacity also change throughout development. Photosynthesis (*A*) often declines over the growing season after leaves have reached full expansion. In some cases this decline in *A* is coordinated with stomatal conductance (*g*_s_) and may limit *A* (Kriedmann *et al.*, 1970; Aslam *et al.*, 1977; Constable and Rawson, 1980; Field and Mooney, 1983; Vos and Oyarzun, 1987; Kositsup *et al.*, 2010).

Leaf hydraulic conductance (*K*_leaf_) is a measure of the efficiency of water transport through the leaf, calculated as water flux through the leaf divided by the water potential driving force. *K*_leaf_ is dynamic, depending on variable aquaporin expression and activation (Maurel *et al.*, 2008) as well as cavitation and embolism refilling in the xylem (Canny, 1997; McCully *et al.*, 1998; Holbrook *et al.*, 2001). The relative impacts of these *K*_leaf_ regulators, particularly embolism and refilling in the xylem, are not fully understood and are under intense study (Wheeler *et al.*, 2013; Scoffoni and Sack, 2014). Because liquid water transport through the leaf is critical to maintain open stomata for CO_2_ acquisition, *K*_leaf_ is strongly linked with *A*, and *K*_leaf_ and maximum photosynthetic capacity are correlated across many species (Brodribb *et al.*, 2005; Sack and Holbrook, 2006).

Given its link with *A* and transpiration, hydraulic conductance is expected to change as leaves age. *K*_leaf_ decreased as leaves age in several evergreen and deciduous tree species (Salleo *et al.*, 2002; Lo Gullo *et al.*, 2005). This decline is, in some cases, linked with photosynthetic parameters (Brodribb and Holbrook, 2003b). Xylem blockage by tyloses that progressively decrease *K*_leaf_ may also be a component of senescence (Cochard and Tyree, 1990; Salleo *et al.*, 2002). However, the dynamics of *K*_leaf_ with leaf age over a growing season have yet to be examined in any herbaceous or annual species, such as *Glycine max* (soybean). Soybean leaves experience much more dramatic microenvironment changes over their lifespan than most tree leaves, as soybean leaves mature in full sun and quickly become shaded and shielded from wind and precipitation by newer leaves above in a dense canopy, and both *A* and *g*_s_ have been shown to decrease as soybean leaves age (Woodward and Rawson, 1976; Reich *et al.*, 1985; Burkey and Wells, 1991). Thus, if a season-long *K*_leaf_ decline exists, it may be even more drastic than in these tree species, and more likely to become limiting to transpiration and thereby photosynthesis.

We hypothesized that *K*_leaf_ and transpiration demand in soybean would remain in balance as leaves aged. This was tested with field-grown and chamber-grown soybean. Because photosynthesis is dependent on water transport through the leaf, a decrease in *K*_leaf_ could indicate a hydraulic component to loss of photosynthetic capacity and leaf senescence. Thus, this study tests whether photosynthesis in older leaves could be hydraulically limited, which would lead to lower total canopy photosynthesis. Although similar studies have been carried out in tree species, the potential limitation of canopy photosynthesis by *K*_leaf_ in older leaves has to our knowledge not yet been examined in any key crop species.

Drought is the main yield-reducing environmental stress facing crops (Boyer, 1982), and rising greenhouse gas concentrations exacerbate this stress by altering global climate patterns, which is expected to increase the frequency of extreme weather events, including drought (Burke *et al.*, 2006; Meehl *et al.*, 2007). During severe drought, tracheary element cavitation is likely to occur at high xylem tensions, reducing *K*_leaf_ (Machado and Tyree, 1994; Meinzer, 2002), although genotypic differences in hydraulic properties among cultivars can affect drought tolerance within a species (Silva *et al.*, 2004; Sadok and Sinclair, 2010a). In some species stomata show a direct decrease in *g*_s_ in response to leaf water potential (Ψ_leaf_), while *K*_leaf_ does not decrease until a threshold Ψ_leaf_ is reached, thereby delaying extensive vessel cavitation (Nardini and Salleo, 2000; Cochard, 2002; Brodribb and Holbrook, 2003a). However, *K*_leaf_ decreases with soil drying in many woody and herbaceous species (Linton and Nobel, 2001; Brodribb and Holbrook, 2003a; Lo Gullo *et al.*, 2003; Blackman *et al.*, 2009; Ferrio *et al.*, 2012). *K*_leaf_ also declined with Ψ_leaf_ across a range of deciduous and evergreen trees and shrubs (Nardini *et al.* 2001; Brodribb and Holbrook 2006; Johnson et al. 2011, 2012; Guyot *et al.* 2012; Bucci *et al.* 2012). Similarly, vapour pressure deficit-induced xylem cavitation resulted in stomatal closure for *Laurus nobilis* L. plants grown in constantly wet soil (Salleo *et al.*, 2000).

As elevated [CO_2_] generally decreases stomatal conductance, it could protect the plant from drought by conserving soil moisture as well as slowing the decrease of Ψ_leaf_ under conditions of limited water or high vapour pressure deficit (Allen *et al.*, 1998, 2003; Leakey *et al.*, 2006). Elevated [CO_2_] has been observed to decrease hydraulic conductance of either whole plants or leaves in several species (Bunce, 1996; Bunce and Ziska, 1998; Domec *et al.*, 2009). However, we have previously observed a lack of *K*_leaf_ acclimation to growth at elevated [CO_2_] for field-grown soybean (Locke *et al.*, 2013) while stomatal conductance consistently decreased (Leakey *et al.*, 2006). Thus, restricted transpiration during growth at elevated [CO_2_] could protect against *K*_leaf_ decline during drought.

In addition to investigating leaf age effects on *K*_leaf_ we tested the hypotheses that soybean *K*_leaf_ will decline during drought and that growth at elevated [CO_2_] will protect leaves from experiencing this decline. These experiments were essential for interpreting *K*_leaf_ data in leaf-age-targeted field experiments during drought years. Drought experiments were conducted both in the field under open air conditions as well as in environmentally controlled growth chambers. Because of the link between leaf hydraulics and gas exchange, measuring the responses of *K*_leaf_ to declines in soil moisture could help predict hydraulic limitation to photosynthesis during drought.

## Materials and methods

### Leaf age experiments

For the leaf age field experiment, soybean cultivar 93B15 (maturity group III) with indeterminate growth (Pioneer Hi-Bred, Johnston, IA, USA) was planted at the Soybean Free Air Concentration Enrichment (SoyFACE) facility in Savoy, IL, USA, on 8 June 2011. The experiment was conducted in six 6 m × 6 m blocks. Soybean was grown in yearly rotation with *Zea mays* (corn) according to standard agricultural practice in central Illinois, USA. Rows were spaced 0.76 m apart, and rows were thinned to one plant per 25cm (52400 plants/ha) when seedlings reached developmental stage VC. Leaves at the third and tenth nodes from the ground were marked with flagging tape tied around their petioles as they emerged.

This field experiment was repeated in 2013, when soybean cultivar 93Y40 (maturity group III) was planted on 6 June. A single plot, measuring 10 m × 15 m, was again thinned to a density of one plant per 25cm (52400 plants/ha). Leaves at the third and tenth nodes were tagged for sampling throughout the growing season.

These field experiments were not irrigated, as is standard practice for soybean farming in central Illinois. This region experienced a progressively worsening drought during the 2011 growing season, and less severe drought but nonetheless progressively drying soils again in 2013. In addition to conducting separate drought experiments, the leaf age experiment was repeated in growth chambers with consistently well-watered plants to confirm that observed results were attributable to leaf age and not to drought.

For the leaf age growth chamber experiment, soybean cultivar PI 154197 (maturity group 00) with determinate growth (Pioneer Hi-Bred) was planted in 14 l pots on 11 January 2013. This cultivar was selected to ensure that the plants did not outgrow the growth chambers. Twelve plants were grown in each of eight growth chambers. Chamber conditions from the time of seed planting were 25 °C, 60% relative humidity and approximately 1000 μmol m^−2^ s^−1^ daytime photosynthetically active radiation (PAR). Plants were fertilized every other day with 50% Long-Ashton solution, amended with 10mM NH_4_NO_3_ (Hewitt, 1966). Leaves at the fifth and eighth nodes from the soil were tagged as they emerged. Plants were rotated within the growth chambers every 2 days and among the growth chambers every 4 days to minimize differential chamber effects.

Vapour pressure deficit was 0.6 kPa lower on average in the growth chambers than at midday above the canopy in the field on all but the final day of field measurements (data not shown). However, older, lower-canopy leaves in the field likely experienced greater uncoupling from atmospheric conditions than lower-canopy leaves in the growth chamber, because growth chamber fans mixed air throughout the entire chamber, keeping lower leaves at a similar temperature and relative humidity as upper canopy leaves even when the leaves were shaded. Thus, direct comparisons between *K*_leaf_ values in the chamber and in the field are not valid, but differences among the growth chamber measurements should represent endogenous patterns in the plant, which are also reflected in field *K*_leaf_ measurements.

*K*_leaf_, Ψ_leaf_ and leaf gas exchange were measured at three developmental stages for leaves at each tagged node: when the leaf was the youngest mature leaf on the plant, at the top of the canopy (stage A); when the leaf was older and shaded but still fully green (stage B); and when the leaf had visibly begun to senesce, considered to be at least 50% yellowed (stage C). Two sets of stage B measurements were taken in the 2013 field experiment, termed B1 and B2. In the chamber experiment measurements were only taken for stages A and B, because a fungal infection in the leaves prevented measurements during senescence. Leaves were assumed to be 10 days old when they reached maturity (stage A); stage B measurements were taken at 19–43 days old in the field experiments and at 23–24 days old in the chamber experiment; and stage C measurements were taken at 54–76 days old in the field experiments.

### Gas exchange

*A* and *g*_s_ were measured with a LI-6400 photosynthesis system equipped with a leaf chamber fluorometer (LI-COR Inc., Lincoln, NE, USA). Measurements were taken between 12:00 and 14:00, as this time typically corresponds to peak *A*. [CO_2_] was 400 ppm and relative humidity was maintained between 50 and 70% in the cuvette for all measurements. Light and block temperature were set to the ambient temperatures experienced by the leaf (Supplementary Table 1). These leaves were tagged after measurement so that the exact same leaves could be sampled for *K*_leaf_ measurement the next morning. Intrinsic water-use efficiency (WUE) was calculated as *A*/*g*_s_.

To determine if declining *A* was merely the result of measurement at decreasing ambient light intensity rather than a down-regulation of photosynthetic capacity, light curves were measured on field-grown leaves at all growth stages in 2013. Leaves were excised before sunrise and their petioles were re-cut under water. *A* was measured over a range of PAR levels from 2000 to 0 µmol m^−2^ s^−1^ for each leaf.

### Leaf water potential

Tissue was harvested for measurement of midday Ψ_leaf_ with thermocouple psychrometers (C30, Wescor Inc., Logan, UT, USA) at the same time as gas exchange measurements were taken. In the field experiment, four leaves were sampled per block; in the chamber experiment, three leaves were sampled per chamber. For each leaf, three 1.2cm discs were removed and sealed into a steel chamber with the thermocouple psychrometer within 15 s of sampling. These chambers were allowed to equilibrate to 25 °C for 2.5–3h before leaf water potential was recorded by a datalogger (CR-7, Campbell Scientific, Logan, UT, USA). Leaf water potentials were then calculated based on a sucrose calibration performed with the psychrometers prior to the experiment.

In the growth chamber experiment, leaf osmotic potential (Ψ_Π_) was measured subsequent to the water potential determinations. Following the Ψ_leaf_ measurements, the steel psychrometer chambers were held in liquid nitrogen for 60 s to lyse the cells and eliminate cell wall turgor pressure. The chambers were then thawed overnight to re-equilibrate to 25 °C. Osmotic potential was recorded by the datalogger. Leaf hydrostatic pressure (Ψ_P_) was calculated as:

$$\Psi_{\text{P}}=\;\Psi_{\text{leaf}}-\;\Psi_{\Pi }$$

### Leaf hydraulic conductance

*K*_leaf_ was measured using the evaporative flux method (Sack *et al.*, 2002; Locke *et al.*, 2013). In this method, water flux through the leaf is measured while the leaf is placed in an environment favourable to transpiration. Leaves were harvested pre-sunrise in the field and before morning growth lights turned on in chamber experiments to ensure that as much embolism refilling as possible had occurred overnight. Leaves were cut with a razor blade at the base of the petiole and immediately placed in distilled water. Petioles were re-cut 2cm shorter under water upon return to the laboratory to remove major cavitation introduced during sampling; 2cm is sufficient to remove introduced embolism, as average vessel length in soybean petioles is less than 1mm (Ghorashy *et al.*, 1969). Leaves which were not sufficiently re-cut typically wilted quickly upon connection to the evaporative flux apparatus and were not included in the analysis. For water flux measurements, petioles were connected to tubing (Tygon R-3693, Saint-Groban Performance Plastics Corporation, Aurora, OH, USA) that led to a reservoir of water on a high-precision balance (± 0.01mg; XS 250, Mettler Toledo, Columbus, OH, USA). Crevices in the petioles were filled with petroleum jelly, and petioles were wrapped with Parafilm (Pechiney Plastic Packaging Company, Chicago, IL, USA) to ensure a tight seal with the tubing. Leaves were illuminated with approximately 700 µmol m^−2^ s^−1^ PAR from a 750W halogen lamp, with a clear water dish directly below the lamp to dissipate heat and a fan blowing on the leaf to reduce the leaf boundary layer. While 700 µmol m^−2^ s^−1^ PAR is usually not photosynthetically saturating for a soybean leaf, it is high enough to stimulate transpiration, and the consistent light level across all measurements ensured that comparisons among leaves are valid. The change in water mass was logged every 30 s by a datalogger (CR1000, Campbell Scientific) simultaneously for four balances, and flow rates were monitored on a single computer. Flow rate typically stabilized in 30–60min, at which point the leaf temperature was recorded (572 Handheld Infrared Thermometer, Fluke Corporation, Everett, WA, USA). Transpiration was sufficient to keep the leaf temperature 1–4 °C lower than ambient temperature (data not shown). Ψ_leaf_ was measured with thermocouple psychrometers as described above. Four psychrometers were used per trifoliate leaf, with three leaf discs per psychrometer chamber. Leaf margins were left intact so that leaves could be photographed, and leaf area was calculated using the freeware ImageJ (http://imagej.nih.gov/ij/).

To calculate *K*_leaf_, flow rate was divided by Ψ_leaf_ of the leaf during the *K*_leaf_ measurement and leaf area. This value was temperature-normalized to account for the viscosity of water, which decreases approximately 2% per 1 °C increase (Yang and Tyree, 1993).

### Drought experiments

A field experiment was conducted in Drought by Rain Interception-FACE (DRI-FACE) plots at the SoyFACE facility in 2010, which independently tested whether drought affects *K*_leaf_. Soybean cultivar 93B15 (Pioneer Hi-Bred) was planted on 27 May 2010 in 38cm row spacing, and CO_2_ fumigation began on 9 June 2010 and continued through senescence. CO_2_ was fumigated with a target of 585 ppm in elevated-[CO_2_] plots as described by Leakey *et al.* (2004). The DRI treatment was implemented with retractable 4.6 m × 9.2 m rain-interception awnings placed within the ambient- and elevated-[CO_2_] plots. The awnings were controlled by a computer and deployed automatically when precipitation was detected by rain sensors and PAR was below 50 µmol m^−2^ s^−1^, as described in detail by Gray *et al.* (2013). This low light threshold ensured that at most 0.05% of growing-season PAR was intercepted by the awnings (Gray *et al.*, 2013). Intercepted rain was diverted 20 m away from the reduced precipitation (RP) plots by gutters. This rain interception treatment resulted in a persistent and progressively increasing disparity between control precipitation (CP) and RP plots over the course of the growing season. Ambient- and elevated-[CO_2_] treatments were applied in a randomized complete block design with four blocks, while precipitation treatments were applied as a split plot within the ambient- and elevated-[CO_2_] plots. *K*_leaf_ was measured on uppermost, fully mature leaves on 3 days over the course of the growing season.

For a drought experiment in growth chambers, soybeans were planted in 14 l pots. Twelve plants were grown in each of four growth chambers (GC-15, Environmental Growth Chambers, Chagrin Falls, OH, USA), with six pots assigned to drought treatment and six treated as controls. Chamber conditions from the time of seed planting were 25 °C, 60% relative humidity and approximately 1000 μmol m^−2^ s^−1^ daytime PAR. Plants were fertilized every other day with 50% Long-Ashton solution, amended with 10mM NH_4_NO_3_ (Hewitt, 1966). Plants were rotated within the growth chambers every 2 days and among the growth chambers every 4 days to minimize differential chamber effects.

Drought treatment was created by withholding water for a period of 4–5 days, until drought plants began to visibly lose turgor. Control pots were watered as normal during dry-down periods. *K*_leaf_, *g*_s_ and Ψ_leaf_ measurements were taken at the end of each dry-down period, and then all plants were re-watered. Dry-down periods were spaced 6 days apart to allow sufficient time for plants to re-hydrate and for a new leaf to mature in well-watered conditions. *K*_leaf_ and midday Ψ_leaf_ were measured as described above.

For the drought growth chamber experiment, *g*_s_ was measured with a steady-state diffusion porometer (model SC-1, Decagon Devices, Pullman, WA, USA). The instrument was allowed to equilibrate to growth chamber conditions for at least 30min before measurement, and measurements were taken on plants while inside the growth chamber. Abaxial conductance was measured on the uppermost fully expanded leaf for 12 plants per treatment.

### Statistical analyses

Differences among growth stages were analysed by repeated measures with the SAS MIXED procedure (SAS Inc., Cary, NC, USA). Node and growth stage were treated as fixed effects, and plots and chambers were considered random blocking effects. Correlations between *K*_leaf_ and gas exchange parameters were tested using the REG procedure.

## Results

### *K*_leaf_ decreases as soybean leaves age

**Fig. 1.**
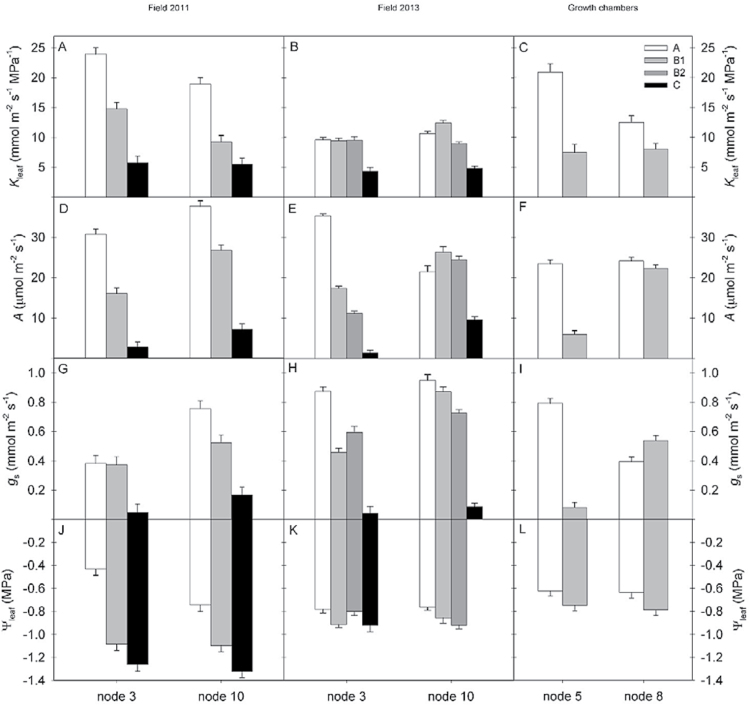
*K*_leaf_, *A*, *g*_s_ and Ψ_leaf_ for each measured leaf stage in all experiments (2011 field experiment, A, D, G, J; 2013 field experiment, B, E, H, K; growth chamber experiment, C, F, I, L). *K*_leaf_ (A–C) was measured with the evaporative flux method for leaves sampled before sunrise, *A* (D–F) and *g*_s_ (G–I) were measured with a LI-COR 6400 Portable Photosynthesis System and midday Ψ_leaf_ (J–L) was measured with thermocouple psychrometers. When two stage B measurements were taken, the earlier stage B measurement is represented by light grey and the later measurement is represented by dark grey. Error bars represent standard error.

**Fig. 2.**
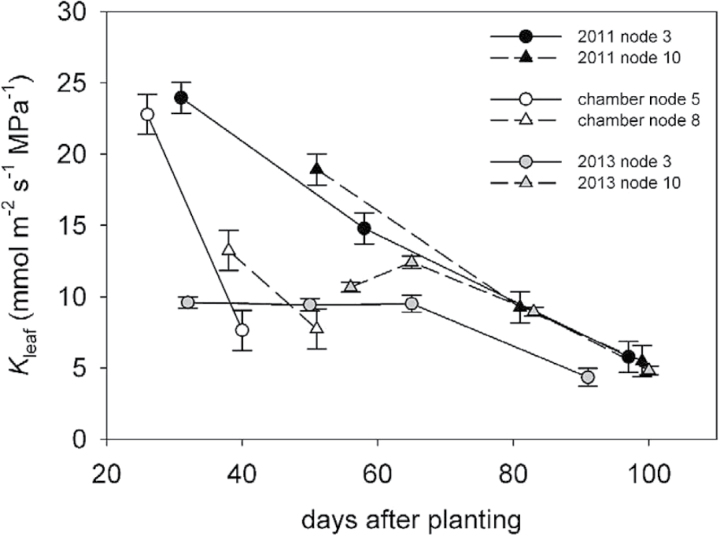
*K*_leaf_ represented by days after planting. 2011 field experiment, closed symbols; growth chamber experiment, open symbols; 2013 field experiment, grey symbols. Circles and triangles denote lower and upper nodes measured. Error bars represent standard error.

In field-grown (Fig. 1A, B) and in chamber-grown (Fig. 1C) soybean, *K*_leaf_ changed as leaves aged (*P* < 0.0001). Leaf stages at measurement are denoted as stage A (youngest, fully mature leaf at the top of the canopy), stage B (older, fully green) and stage C (visibly senescent). The decrease in *K*_leaf_ appeared to be consistent for field-grown plants in 2011 (Fig. 1A) and chamber-grown plants (Fig. 1C), while for field-grown plants in 2013 a decrease was either not apparent until the end of the season, as for node 3, or was interrupted by peak *K*_leaf_ during stage B, as for node 10 (Fig. 1B). For field-grown soybean, *K*_leaf_ decreased by 56–76% from stage A to stage C. *K*_leaf_ decreased more rapidly over time for the determinate cultivar in the growth chamber experiment than the indeterminate cultivars grown in the field (Fig. 2), consistent with the shorter maturity group (00) grown in the chamber experiments versus the field experiments (maturity group III). *K*_leaf_ at leaf stage A was lower at upper canopy nodes in the 2011 field experiment and in growth chambers, but this was not observed in the 2013 field experiment. While *K*_leaf_ values at stages B and C were comparable across experiments all three experiments, despite different genotypes and growing conditions, *K*_leaf_ at stage A was substantially lower in the 2013 field experiment, causing the less consistent season-long decrease in *K*_leaf_.

### Photosynthetic capacity decreased coordinately with *K*_leaf_ as leaves aged

**Fig. 3.**
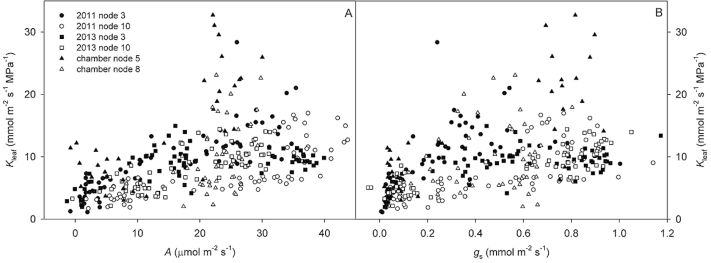
*K*_leaf_ and *A* (A) and *K*_leaf_ and *g*_s_ (B) for all leaves measured across the growing season. *A* and *g*_s_ were measured at midday, and maximum *K*_leaf_ was measured for the exact same leaves sampled before sunrise the next morning. Measurements at all leaf ages are included for the experiment/node combinations shown. Regressions were calculated separately for each experiment/node, and the correlation statistics for *K*_leaf_/*A* and *K*_leaf_/*g*_s_ relationships at each node are listed in Table 1.

**Fig. 4.**
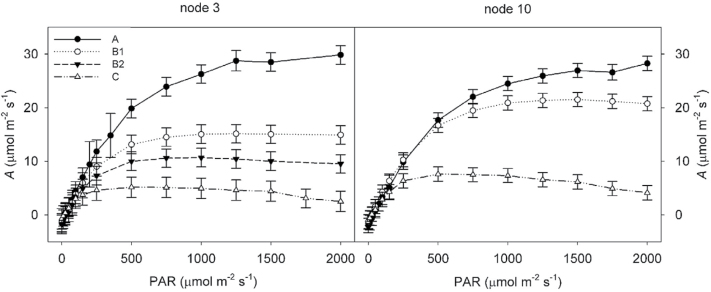
Changes in leaf photosynthetic capacity as leaves age. *A* was measured at a range of PAR from 2000 to 0 µmol m^−2^ s^−1^ for leaves at each stage. Leaves were excised before sunrise and light-acclimated before measurement. Measurements were taken at stage A (closed circles), stage B/B1 (open circles), stage B2 (closed triangles), and stage C (open triangles). Error bars represent standard error.

*A* was measured at midday, around the time of peak diurnal photosynthesis. This evaluation of maximum photosynthesis along with maximum (pre-sunrise) *K*_leaf_ allows for the examination of season-long trends in leaf hydraulic and photosynthetic capacities. *A* also decreased consistently as leaves aged in all three experiments (*P* < 0.0001), except for at node 10 in the 2013 field experiment (Fig. 1D–F). The pairwise decrease was small (*P* = 0.20) for leaves at node 8 in the growth chamber experiment, which did not become shaded by younger leaves as they aged as a result of determinate growth. *K*_leaf_ and *A* were significantly correlated across leaf ages for all nodes measured in all experiments, although strength of these relationships varied widely among nodes, with *R* ^2^ varying from 0.13 to 0.62 (Table 1, Fig. 3A). In 2013, the light response of photosynthesis was measured at each leaf stage to determine if lower photosynthetic rates in older leaves is simply the result of low light beneath the canopy not maximizing photosynthetic capacity, or if photosynthetic capacity was actually down-regulated in older leaves. Light saturated photosynthesis was found to decrease consistently as the leaves aged for both node 3 (Fig. 4A) and node 10 (Fig. 4B). Leaves at node 3, stage A, reached a maximum photosynthetic rate of 29.9 µmol m^−2^ s^−1^ at 2000 µmol m^−2^ s^−1^ PAR, while maximum photosynthesis for the same leaves at stage C was only 5.2 µmol m^−2^ s^−1^ at 500 µmol m^−2^ s^−1^ PAR.

**Table 1.** Correlation coefficients for the relationships between K_leaf_ and A or g_s_ across the entire growing season for each node in every experiment

	Node	*R* ^2^	
		*K*_leaf_ vs *A*	*K*_leaf_ vs *g*_s_
Field 2011	3	0.61***	0.30***
	10	0.62***	0.53***
Field 2013	3	0.23***	0.34***
	10	0.59***	0.71***
Growth chamber	5	0.51***	0.59***
	8	0.13*	n.s.

Leaf gas exchange was measured at midday, and these exact same leaves were sampled for *K*_leaf_ measurement before sunrise the next morning. Asterisks indicate the significance of the correlation (*P < 0.05, ***P < 0.0001; n.s., not significant).

Stomatal conductance decreased overall as leaves aged for field-grown soybean in 2011 (*P* < 0.0001, Fig. 1G). For chamber-grown soybean (Fig. 1I), however, *g*_s_ decreased 90% from stage A to stage B for node 5 (pairwise *P* < 0.0001), while *g*_s_ increased 36% from stage A to stage B for node 8 (pairwise *P* < 0.01). The increase or decrease in *g*_s_ between specific stages did not as closely follow the patterns of *K*_leaf_ as those of *A* did, and the correlations between *K*_leaf_ and *g*_s_ were thus sometimes weaker than correlations between *K*_leaf_ and *A* (Table 1). In field-grown soybean, *K*_leaf_ correlated with *g*_s_ for both node 3 (*R* ^2^ = 0.30) and node 10 (*R* ^2^ = 0.53), but these correlations were not as strong as those between *K*_leaf_ and *A* (Table 1, Fig. 3B). *K*_leaf_ and *g*_s_ were not correlated for chamber-grown soybean (Table 1, Fig. 3B).

WUE was calculated from gas exchange data and did not change across experiments as leaves aged in either field- or chamber-grown soybean. Contrasts within nodes showed that WUE differs among stages (*P* < 0.0001 for all nodes), but the direction of these changes was not consistent and the significance does not hold across either experiment. *K*_leaf_ and WUE were not correlated for field- or chamber-grown soybean (data not shown).

### Ψ_leaf_ declines as soybean leaves age and is driven by decreasing osmotic potential

Ψ_leaf_ decreased as leaves aged for field-grown soybean in 2011 (*P* < 0.0001) and chamber-grown plants (*P* < 0.0001), but it did not change significantly as leaves aged in the 2013 field experiment (Table 2, Fig. 1J–L). From stage A to stage B in the field experiment, Ψ_leaf_ decreased 0.65MPa at node 3 and 0.35MPa at node 10. From stage A to stage C, Ψ_leaf_ decreased 0.83MPa at node 3 and 0.58MPa at node 10. For chamber-grown soybeans, Ψ_leaf_ decreased 0.13MPa at node 5 and 0.15MPa at node 8. In the 2013 field experiment and the growth chamber experiment, Ψ_Π_ and Ψ_P_ were also measured to determine what was driving changes in Ψ_leaf_. In growth chambers, Ψ_Π_ decreased as leaves aged as did Ψ_leaf_, while Ψ_P_ remained steady across the growing season. Ψ_Π_ decreased by 0.11MPa at node 5 and by 0.19MPa at node 8 (*P* < 0.001). In the 2013 field experiment, osmotic potential also changed significantly as leaves aged, but the decrease was only steady at node 10, while Ψ_Π_ actually increased from stages B1 to B2 and B2 to C at node 3 (Table 2).

**Table 2.** Mean values for leaf water potential, osmotic potential and turgor pressure ± standard error for the 2013 field experiment and the growth chamber experiment

Experiment	Node	Stage	Water potential	Osmotic potential	Turgor pressure
Field 2013	3 *Ψ_Π_	A	−0.78±0.03	−1.00±0.03	0.22±0.03
		B1	−0.91±0.03	−1.08±0.04	0.22±0.03
		B2	−0.80±0.04	−0.97±0.04	0.17±0.04
		C	−0.92±0.06	−0.93±0.11	0.09±0.09
	10 *Ψ_Π_	A	−0.76±0.03	−0.96±0.03	0.20±0.02
		B1	−0.85±0.05	−0.99±0.05	0.14±0.03
		B2	−0.92±0.03	−1.08±0.05	0.16±0.04
Growth chambers	5	A	−0.62±0.04	−0.79±0.05	0.17±0.03
		B	−0.75±0.05	−0.90±0.05	0.15±0.04
	8	A	−0.63±0.05	−0.93±0.05	0.29±0.04
		B	−0.79±0.05	−1.12±0.05	0.35±0.04

Asterisks indicate a significant change (*P* < 0.05) in a parameter for a particular node.

### Soybean *K*_leaf_ does not acclimate to drought

**Fig. 5.**
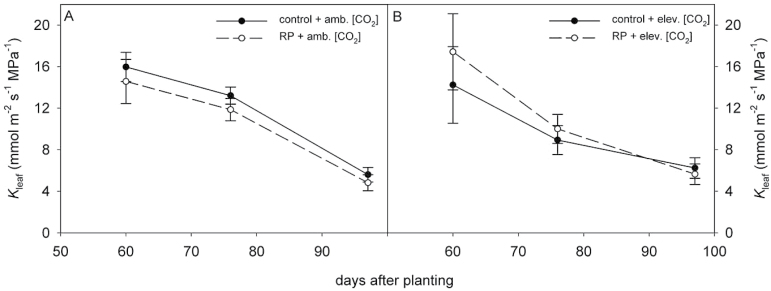
*K*_leaf_ for plants grown in RP plots (closed circles) compared to plants grown in control precipitation plots (open circles). Precipitation treatments were conducted under both ambient [CO_2_] (385 ppm; A) and elevated [CO_2_] (585 ppm; B); panels are separated for clarity. *K*_leaf_ was measured on uppermost, fully expanded leaves sampled before sunrise in the field.

In the field, DRI awnings intercepted 41% of growing-season precipitation, resulting in soil moisture decreases of up to 50%, as reported in detail by Gray *et al.* (2013). *K*_leaf_ for field-grown soybean decreased significantly over the course of the growing season when measured on youngest fully expanded leaves (*P* < 0.0001) (Fig. 5). Pre-dawn *K*_leaf_ decreased from an average across treatments of 15.4 mmol m^−2^ s^−1^ MPa^−1^ at 60 days after planting to 10.8 and 5.9 mmol m^−2^ s^−1^ MPa^−1^ at 76 and 97 days after planting (Fig. 5A, B). Therefore, treatment effects were analysed separately for each measurement day. *K*_leaf_ was not affected by persistent drought on any measurement day, and this was the case at both ambient (Fig. 5A) and elevated [CO_2_] (Fig. 5B). *K*_leaf_ was lower for elevated-[CO_2_] plants than for ambient-[CO_2_] plants on day 76 (*P* = 0.0208; Fig. 5A, B), but [CO_2_] did not affect *K*_leaf_ on the other two measurement days. On day 76, the difference in *K*_leaf_ between [CO_2_] treatments was primarily driven by the control precipitation plots, which had the highest *K*_leaf_ of all four treatments at ambient [CO_2_] and the lowest *K*_leaf_ of all four treatment combinations at elevated [CO_2_], whereas there was no difference between *K*_leaf_ for ambient and elevated [CO_2_] in drought plants.

**Fig. 6.**
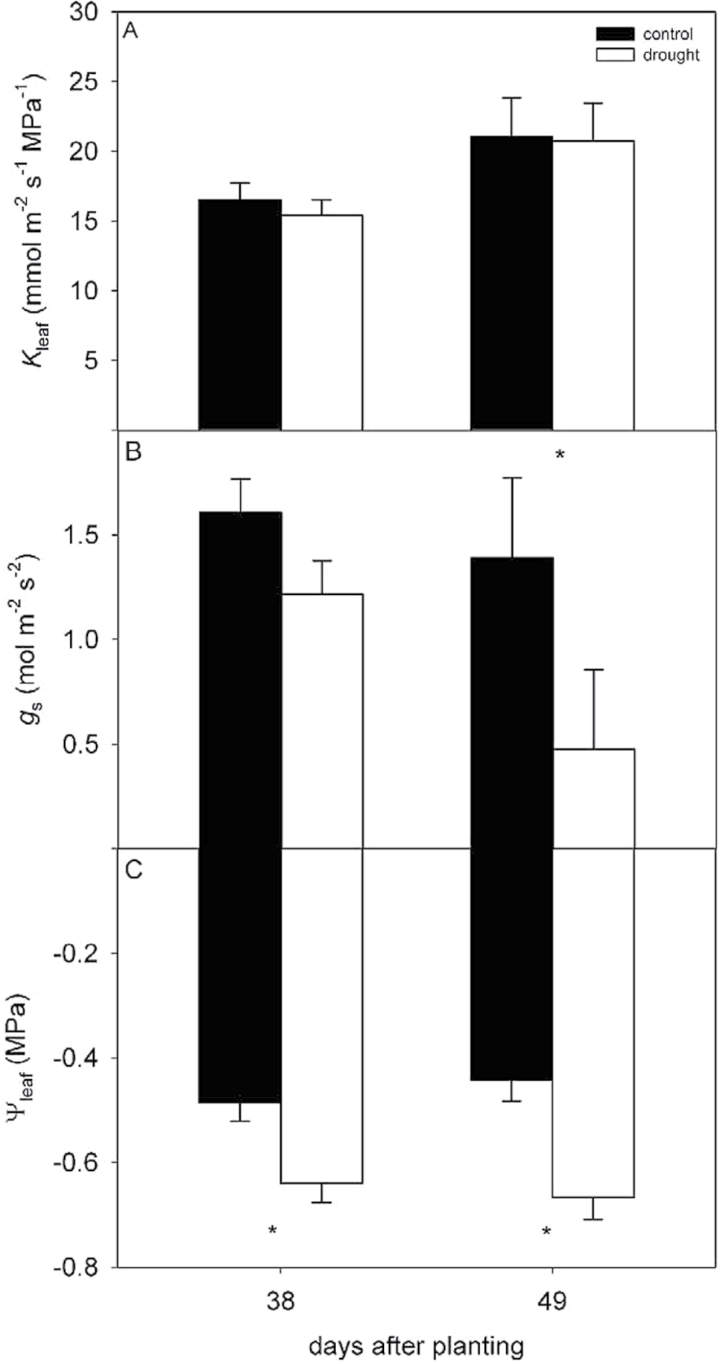
Pre-sunrise *K*_leaf_ (A), midday *g*_s_ (B) and midday Ψ_leaf_ (C) for drought and control plants grown in growth chambers. Measurements were taken at the end of a 4–5 day period during which water was withheld from drought treatment plants, while control plants were watered as usual. All measurements were made on uppermost, fully expanded leaves. *K*_leaf_ was measured with the evaporative flux method, *g*_s_ was measured with a porometer and Ψ_leaf_ was measured with thermocouple psychrometers. Asterisks indicate a significant difference between drought and control treatments. Error bars represent standard error.

In the growth chamber experiment, by withholding water for 4–5 days during each drought cycle, volumetric soil moisture was decreased by an average of 62% on day 38 and 66% on day 49 in the drought pots compared to control pots. This soil moisture deficit was sufficient to significantly decrease Ψ_leaf_ by 33% on day 38 (*P* = 0.0213) and 50% on day 49 (*P* = 0.0546) (Fig. 6C). Stomatal conductance (*g*_s_) was 24 and 66% lower on days 38 and 49 in drought than in control plants, a response to soil drying and Ψ_leaf_ (Fig. 6B). The *g*_s_ decrease was only significant at α = 0.01 on day 49 (*P* = 0.0661). However, despite declines in soil moisture, Ψ_leaf_ and *g*_s_, *K*_leaf_ in drought plants was not different from *K*_leaf_ in control plants on either day (*P* = 0.37 and *P* = 0.95) (Fig. 6A), although *K*_leaf_ for both treatments was higher on day 49 than on day 38.

## Discussion

Field and growth chamber data both showed a trend of decreasing *K*_leaf_ as soybean leaves age, although the decrease was not always consistent over the course of the plant’s lifespan. Similar *K*_leaf_ behaviour observed in well-watered growth chamber plants as compared to field-grown plants and the absence of a *K*_leaf_ response to deliberately manipulated soil moisture in separate experiments support the conclusion that the observed declines in *K*_leaf_ in leaf age experiments were linked to leaf aging rather than varying soil water availability in the field. *K*_leaf_ was as unresponsive to short, sudden drought periods when grown in pots as it was to prolonged drought in the field. The drought treatments imposed in the chamber experiments were substantial enough to decrease both Ψ_leaf_ and *g*_s_, supporting the conclusion that *K*_leaf_ in soybean does not acclimate to protect against cavitation or loss of *g*_s_ during drought.

The observed decrease in *K*_leaf_ as leaves age may be a result of down-regulation or inactivation of aquaporin proteins in living cells or of xylem blockages, such as emboli or tyloses. The lower Ψ_leaf_ in senescing leaves compared with young leaves observed in both field-grown and chamber-grown soybean in this experiment would increase the risk of cavitation in xylem of older leaves (Tyree and Sperry, 1989). There is evidence that repeated cycles of cavitation and refilling over the course of the growing season can weaken xylem pit membranes, making the xylem more vulnerable to cavitation over time (Sperry *et al.*, 1991; Hacke *et al.*, 2001). This vulnerability increase in conjunction with decreasing water potential has also been implicated in the decline of *K*_leaf_ in *Rhedera trinervis* and *Calycophyllum candidissimum* during leaf senescence (Brodribb and Holbrook, 2003b). By this mechanism, the lower Ψ_leaf_ observed in older leaves could lead to a build up of emboli, which the leaf becomes unable to completely refill overnight as the growing season progresses. An increasing number of emboli may also allow the formation of tyloses, which have been implicated in leaf abscission (Sexton and Roberts, 1982). Careful measurements of aquaporin expression and activity, as well as xylem imaging, could illuminate the mechanism(s) by which *K*_leaf_ decreases in the long-term.

*A* decreased similarly to *K*_leaf_ as leaves aged. Although lower *A* in older leaves could be attributed simply to lower light intensity within the canopy, reductions in *A* with leaf age are common even at near-saturating irradiance (Vos and Oyarzun, 1987). Light response curves confirmed that photosynthetic capacity was reduced in older soybean leaves (Fig. 3). All *K*_leaf_ measurements were taken at the same near-saturating light intensity, so the observed correlations between *K*_leaf_ and *A* suggest that the long-term regulation of these parameters is functionally coordinated in soybean. The varying strengths of the correlations between *K*_leaf_ and *A* as compared to *K*_leaf_ and *g*_s_ indicate that *A* may respond to hydraulic capacity in a manner that is not solely mediated by a stomatal limitation to CO_2_ intake.

*K*_leaf_ for the uppermost fully expanded leaves (stage A) was usually lower when the plants were older, as observed in the field-based leaf age experiments and in the DRI-FACE experiment (Figs 2 and 6). However, such consistent patterns were not observed for *A* and *g*_s_, leading to variability in the slope of the relationships between *K*_leaf_ and *A* as well as *K*_leaf_ and *g*_s_ (Fig. 3). The differences in *A* for leaves at the same growth stage, but different nodes, appeared to be driven by changes in *g*_s_ rather than *K*_leaf_ (Fig. 1D–I). This suggests that *A* is likely not limited by *K*_leaf_, except possibly during senescence. The 2011 late-season increase in upper-canopy *A* is consistent with reports of whole-plant photosynthesis peaking during the seed filling period, when sink strength is greatest (Wells, 1991), although this pattern was not observed in 2013.

While canopy WUE frequently decreases over multiple growing seasons as tree stands age (Köstner *et al.*, 2002), WUE did not change consistently as leaves age in a single growing season for soybean (data not shown), which is consistent with observations in *Gossypium hirsutum* and *Lepechinia calycina* (Constable and Rawson, 1980; Field and Mooney, 1983). Although *K*_leaf_ was correlated separately with both *A* and *g*_s_ in soybean, decreases in *K*_leaf_ over the growing season apparently do not function to maintain a balance between water lost and carbon gained. The variability in WUE support the finding that *K*_leaf_ is sometimes more strongly coordinated with *A* than with *g*_s_ in soybean; both suggest that *g*_s_ can be regulated in a more transient manner by microenvironment, whereas *A* is more tightly controlled by gradual biochemical acclimation to overall shifts in microenvironment as leaves age.

Variation in the coordination between *K*_leaf_ and *A* as compared to *K*_leaf_ and *g*_s_ may further be attributable to the different degrees of leaf uncoupling from the atmosphere experienced by field-grown plants and chamber-grown plants. In the field, older, lower-canopy leaves become greatly uncoupled from the atmosphere after canopy closure, experiencing calmer, moister air in addition to lower light intensity. This greatly decreases transpiration demand in addition to triggering light acclimation of photosynthesis. In growth chambers, however, plants were grown alone in pots, resulting in a much lower effective planting density, so lower canopy leaves were both not as shaded and less uncoupled from the ‘atmosphere’ than upper leaves.

Ψ_Π_ decreased as leaves aged in growth chambers, but not consistently in the field. Although declines in Ψ_Π_ over the course of the growing season have been observed in some evergreen tree species and woody understorey species, these decreases were linked to drought conditions (Sobrado, 1986; Ishida *et al.*, 1992). As the chamber-grown plants for which this decline was most pronounced were always well-watered, osmoregulation could be a mechanism for soybean leaves to maintain turgor when *K*_leaf_ declines in older leaves. This would facilitate continued, if decreased, *A* in older leaves.

Because *K*_leaf_ was measured for leaves sampled before sunrise (or before growth chamber lights turned on for the day), any emboli that may have formed in xylem during the previous day had likely refilled overnight (McCully *et al.*, 1998; Yang *et al.*, 2012). Thus, the observed *K*_leaf_ values represent the maximum *K*_leaf_ as determined by venation architecture and mesophyll pathways, and any difference in *K*_leaf_ between treatments in the drought experiments would have been due to acclimation of the leaves to soil moisture conditions rather than transient midday decline in *K*_leaf_ by refillable embolism. A decrease in maximum *K*_leaf_ could protect the leaf from daytime *K*_leaf_ decrease due to embolism (Sadok and Sinclair, 2010b). Because no acclimation was observed, however, soybean likely does not have phenotypic plasticity to respond to soil moisture conditions either by adjusting vein density during leaf development or by aquaporin regulation in mature leaves. This is similar to the lack of *K*_leaf_ plasticity we have previously observed for soybean in response to growth at elevated [CO_2_] and temperature (Locke *et al.*, 2013). Because maximum *K*_leaf_ is the same for plants in both control and RP treatments while soil moisture is decreased, plants in the RP plots are likely more vulnerable to cavitation during transpiration, particularly when vapour pressure deficit is high during the middle of the day. Diurnal cycles of embolism and vessel refilling driven by vapour pressure deficit are thought to occur frequently, and low soil moisture would increase midday tension in the xylem even further, causing more cavitation (Hacke *et al.*, 2001). The inability of *K*_leaf_ to acclimate to decreasing soil moisture may leave soybean leaves more vulnerable to cavitation during peak midday transpiration demand. This vulnerability could contribute to the observed depression in midday *g*_s_ in chamber-grown, water-stressed soybean leaves. The decrease in *g*_s_ without maximum *K*_leaf_ acclimation suggests that stomatal sensitivity to dry soil protects against hydraulic failure in soybean (Brodribb and Holbrook, 2004).

The reduced precipitation treatment left RP plots with rainfall levels equivalent to some of the driest years of the last 60 in the Champaign, IL area. Although the lowest average soil moisture achieved during dry-down periods for chamber-grown soybean was about 30% v/v, which is typically well above the permanent wilting point, there was enough variation in drought treatment pots that some drought treatment plants were already visibly losing leaf turgor. Furthermore, the pots were watered with fertilizer that had a high solute concentration, which likely made root water uptake more difficult for plants even at a soil volumetric water content that would be sufficient in central Illinois soil.

Contrary to previous findings, there was a slight difference in *K*_leaf_ between ambient- and elevated-[CO_2_] plants on one measurement day, 76 days after planting (Fig. 6), but this effect disappeared when the field data from all three measurement days were analysed as a repeated measures model. At this time in the growing season, there was a slight difference in soil moisture between ambient- and elevated-[CO_2_] plots that could have contributed to this difference in *K*_leaf_ (Gray *et al.*, 2013), although this short-lived difference likely had no impact on photosynthesis or water use on timescale of the whole growing season.

The effects of drought on *K*_leaf_ in a major field-grown crop had not been previously examined, and, taken together, these field and chamber experiments suggest that *K*_leaf_ in soybean does not acclimate to drought. Because maximum *K*_leaf_ does not adjust to decreased soil moisture conditions, soybean leaves may be extra vulnerable to cavitation and loss of *K*_leaf_ during daytime transpiration when grown in drought conditions. Thus, inability of *K*_leaf_ to acclimate to drought has the potential to limit stomatal conductance and photosynthesis under severe soil moisture deficit.

Studies with other species suggest that hydraulic failure throughout the plant initiates the process of leaf senescence and shedding (Rood *et al.*, 2000; Salleo *et al.*, 2002; Brodribb and Holbrook, 2003b). While it cannot be concluded from these data if *K*_leaf_ decline in soybean triggers photosynthetic decline and senescence, these results show that hydraulic decline, accompanied by gradual decreases in *A* and leaf water status, is a part of leaf maturation and senescence in soybean. If *K*_leaf_ is limiting *A* in older leaves, then an improvement in hydraulic maintenance could have the potential to increase canopy-level photosynthesis, which is a critical target for crop yield improvement (Zhu *et al.*, 2010).

## Supplementary material

Supplementary material is available at *JXB* online.

Supplementary Table 1 Measurement dates and LI-6400 settings for midday gas exchange measurements. Light and temperature for gas exchange measurements were based on ambient weather conditions. *A*, *g*_s_ and Ψ_leaf_ were measured at midday on the dates shown, and leaves were sampled before sunrise the following morning for *K*_leaf_ measurements.

Supplementary Data

## Abbreviations:

A: photosynthesis
DRI: Drought by Rain Interception
*g*_s_: stomatal conductance
*K*_leaf_: leaf hydraulic conductance
Ψ_leaf_: leaf water potential
Ψ_Π_: leaf osmotic potential
Ψ_P_: leaf hydrostatic potential
PAR: photosynthetically active radiation
RP: reduced precipitation
WUE: sisintrinsic water-use efficiency.
