# Supplementary material for: Leaf hydraulic conductance declines in coordination with photosynthesis, transpiration and leaf water status as soybean leaves age regardless of soil moisture
Source: J Exp Bot. 2014 Oct 3;65(22):6617–27. doi: 10.1093/jxb/eru380 (PMC4246190; doi:10.1093/jxb/eru380)
Supplement: Supplementary Data [file supp_65_22_6617__index.html]

Leaf hydraulic conductance declines in coordination with photosynthesis, transpiration and leaf water status as soybean leaves age regardless of soil moisture — Leaf hydraulic conductance declines in coordination with photosynthesis, transpiration and leaf water status as soybean leaves age regardless of soil moisture — Supplementary Data 

# Leaf hydraulic conductance declines in coordination with photosynthesis, transpiration and leaf water status as soybean leaves age regardless of soil moisture

## Supplementary Data

Data files

**Files in this Data Supplement:**

- Supplementary Data - Supplementary Data
