# Supplementary material for: Leaf hydraulic conductance declines in coordination with photosynthesis, transpiration and leaf water status as soybean leaves age regardless of soil moisture
Source: J Exp Bot. 2014 Oct 3;65(22):6617–27. doi: 10.1093/jxb/eru380 (PMC4246190; doi:10.1093/jxb/eru380)
Supplement: Supplementary Data [file supp_eru380_jexbot128470_file001.pdf]

Table S1. Measurement dates and LI-6400 settings for mid-day gas exchange measurements. Light and temperature for gas exchange measurements were based on ambient weather conditions. A, g_s, and Ψ_{leaf} were measured at mid-day on the dates shown, and leaves were sampled before sunrise the following morning for K_{leaf} measurements.

Node	Stage	Date	PAR (μmol m^{-2} s^{-1})	Block temperature (°C)
3	A	8 July 2011	1700	30.0
3	B	4 August 2011	875	29.0
3	C	12 September 2011	300	28.0
10	A	28 July 2011	1600	31.0
10	B	27 August 2011	1500	28.0
10	C	14 September 2011	300	20.0
3	A	7 July 2013	2500	28.0
3	B1	25 July 2013	500	28.0
3	B2	9 August 2013	300	30.0
3	C	4 September 2013	300	27.0
10	A	31 July 2013	1000	24.0
10	B1	9 August 2013	500	30.0
10	B2	27 August 2013	1000	31.0
10	C	13 September 2013	1000	24.0
5	A	5 February 2013	1000	25.0
5	B	19 February 2013	300	25.0
8	A	17 February 2013	1000	25.0
8	B	2 March 2013	1000	25.0
